# ESPO and Health Sciences Section Symposium: Conducting Clinical Trial Research During the COVID-19 Pandemic: Transforming Research Practice for the Future

**DOI:** 10.1093/geroni/igab046.389

**Published:** 2021-12-17

**Authors:** Brianna Morgan, An Nguyen, An Nguyen

## Abstract

The COVID-19 pandemic caused significant disruptions for people and institutions across healthcare settings. Clinical trials are an important research tool to test interventions in real-world healthcare settings and provide high quality evidence that supports older adults’ longevity and wellness. Clinical trialists must consider how to account for unpredictable and ever-changing environmental contexts. The COVID-19 pandemic is a specific example of a changing context that impacted all stages of the clinical trials process from planning, to administration, and outcomes. Reflecting on ways clinical trialists navigated their studies during the COVID-19 pandemic may unlock opportunities to design flexible clinical trials that meet the needs of older adults in real-world environments. This symposium highlights five clinical trials for older adults that occurred during the COVID-19 pandemic. Dr. Carpenter will discuss lessons learned in implementing a palliative care intervention in nursing homes. Brianna Morgan will describe the pivots needed to complete a clinical trial testing an advance care planning website for nursing home residents with dementia. Dr. Nuckols will describe obstacles and opportunities to implementing a randomized controlled trial on hospital nursing units, including implications for medication safety. Dr. Pevnick will highlight barriers and facilitators to implementing a pharmacist-led intervention to reduce hospital readmissions. Dr. Stark will share novel procedures for conducting clinical trials in the community that reduce burden for older adult participants while maintaining fidelity. Presenters will address practice transformations that researchers can bring forward to design flexible clinical trials that meet the needs of older adults in different healthcare contexts.

